# A Low-Power and Portable Biomedical Device for Respiratory Monitoring with a Stable Power Source

**DOI:** 10.3390/s150819618

**Published:** 2015-08-11

**Authors:** Jiachen Yang, Bobo Chen, Jianxiong Zhou, Zhihan Lv

**Affiliations:** 1 School of Electronic Information Engineering, Tianjin University, 92 Weijin Road, Tianjin 300072, China; E-Mails: yangjiachen@tju.edu.cn (J.Y.); zhoujx@tju.edu.cn (J.Z.); 2 Shenzhen Institutes of Advanced Technology(SIAT), Chinese Academy of Science, Shenzhen 518055, China; E-Mail: lvzhihan@gmail.com

**Keywords:** respiratory monitoring, low power, portability, modulation technology, flow measurement, CO_2_ concentration monitoring

## Abstract

Continuous respiratory monitoring is an important tool for clinical monitoring. Associated with the development of biomedical technology, it has become more and more important, especially in the measuring of gas flow and CO${}_{2}$ concentration, which can reflect the status of the patient. In this paper, a new type of biomedical device is presented, which uses low-power sensors with a piezoresistive silicon differential pressure sensor to measure gas flow and with a pyroelectric sensor to measure CO${}_{2}$ concentration simultaneously. For the portability of the biomedical device, the sensors and low-power measurement circuits are integrated together, and the airway tube also needs to be miniaturized. Circuits are designed to ensure the stability of the power source and to filter out the existing noise. Modulation technology is used to eliminate the fluctuations at the trough of the waveform of the CO${}_{2}$ concentration signal. Statistical analysis with the coefficient of variation was performed to find out the optimal driving voltage of the pressure transducer. Through targeted experiments, the biomedical device showed a high accuracy, with a measuring precision of 0.23 mmHg, and it worked continuously and stably, thus realizing the real-time monitoring of the status of patients.

## 1. Introduction

Recently, with the development of biosensor technology, biomedical devices have been widely developed for health monitoring, and health monitoring, especially respiratory monitoring, which mainly includes gas flow and CO${}_{2}$ concentration, has been increasingly emphasized [1]. End-tidal carbon dioxide (P${}_{E}$${}_{T}$CO${}_{2}$) monitoring has been listed as one of routine monitoring items by the American Society of Anesthesiologists (ASA) [2], and gas flow monitoring provides more valuable information about patients and accurately delivers tidal volumes to a critically sick patient [3]. For respiratory monitoring, we have done the research to measure the CO${}_{2}$ concentration and achieved the staged research result in our works [4,5,6,7]. However, in most previous works, few studies focused on the monitoring of the gas flow and CO${}_{2}$ concentration simultaneously [8,9,10]. Additionally, there exist some difficulties, including size limits, the requirement for monitors to work continuously for a long time and the requirement for a stable power source. Therefore, it is urgent to use small portable and stable biomedical device that can be attached to the patient for clinical applications [11,12,13,14,15].

**Figure 1:**
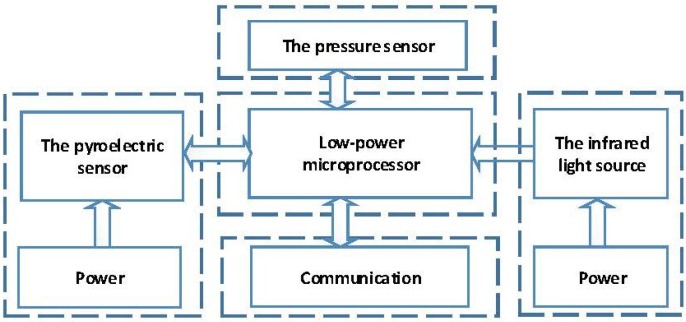
Framework of the detection system.

In this paper, a biomedical device is presented that can measure the gas flow and CO${}_{2}$ concentration simultaneously. The gas flow monitoring is based on the Bernoulli law and is achieved by measuring differential pressure with a pressure sensor. The CO${}_{2}$ concentration is based on the principle of light absorption with a pyroelectric sensor in the infrared region, which is known as non-dispersive infrared absorption (NDIR) [16,17,18,19]. There are two types of techniques used for respiratory monitoring: mainstream and sidestream. Compared to the sidestream device, the mainstream device has the advantages of fast response time, sensitive measurement and high accuracy [5,20,21,22]. Besides, it also meets the requirement of portability. Figure 1 shows the framework of the detection system.

The zero output of the pressure sensor drifts, which will influence the monitoring of gas flow. The sensor data selected may be inaccurate due to the power limitation [23,24], and it was found that there is a relation between the drift and the drive of the pressure sensor. Therefore, we took the power of the sensor into consideration and used mathematical statistics to determine the best drive. The pyroelectric sensor and infrared light source were supplied with power separately, and the infrared light source was modulated to avoid the fluctuation at the trough of waveform [25,26]. In addition, improving related circuits can help avoid the noise of signals.

The primary purpose of the current paper is to present a low-power and portable biomedical device.

## 2. Innovative Respiratory Monitoring System

### 2.1. Design of the Biomedical Module

**Figure 2:**
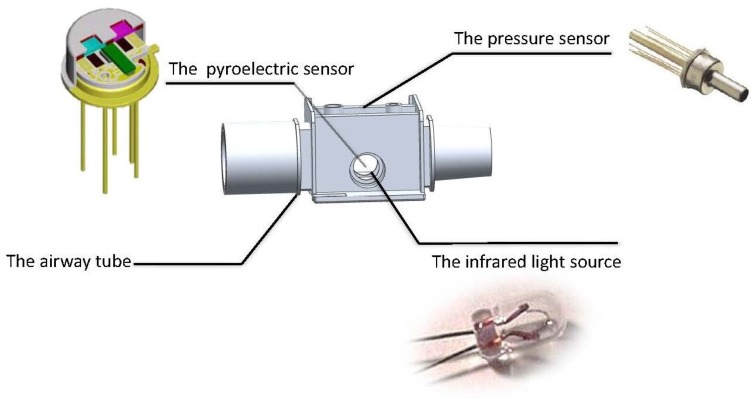
Biomedical module.

The whole biomedical device mainly consists of the respiratory airway tube, the gas flow measurement module and the CO${}_{2}$ concentration measurement module, which are integrated to form a portable biomedical device, as shown in Figure 2.

**Figure 3:**
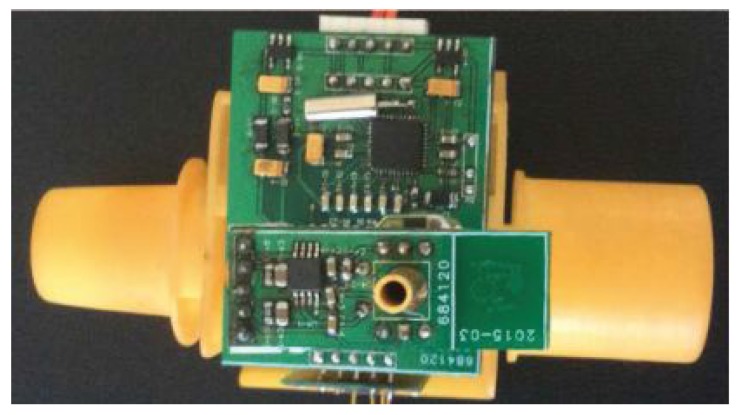
Equipment for respiratory monitoring.

Generally, a biomedical device for health monitoring needs to achieve specific functions under strict medical criteria and significant hardware resource limitations [1]. More specifically, the weight and size of the device need to be small. Given these requirements, an airway tube was designed at the smallest size possible. The size of the respiratory circuits is determined by the structure of the airway tube, as shown in Figure 3.

### 2.2. Gas Flow Measurement Module

The flow meter adopted in the paper is an orifice meter, and the gas flow is measured by the method that is used to measure the differential pressure based on the Bernoulli law:

(1) $$\frac{1}{2}\rho v_{A}^{2}+p_{A}=\frac{1}{2}\rho v_{B}^{2}+p_{B}$$

where *ρ* is the density of air, $\nu_{A}$ and $\nu_{B}$ are the velocities of the airflow at the two different points and $p_{A}$ and $p_{B}$ are the magnitude of the pressure at two different points.

**Figure 4:**
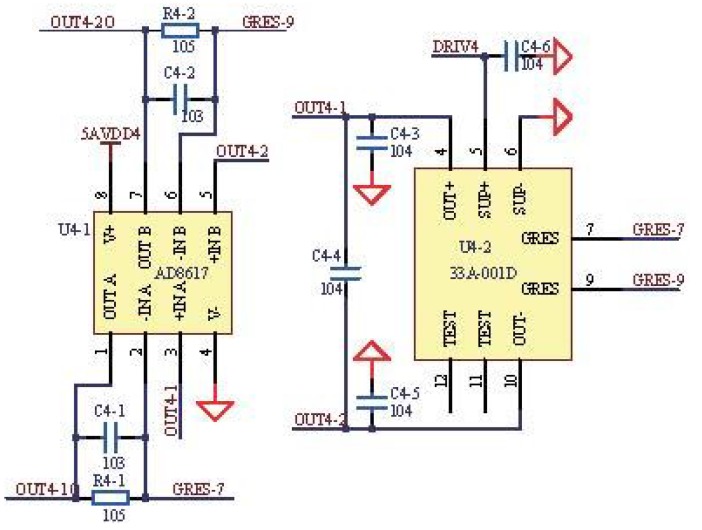
Application circuit of the pressure sensor.

The temperature compensation, piezoresistive silicon pressure sensor was employed in the system, which could achieve a higher voltage sensitivity and lower temperature sensitivity and provide a sensitivity interchangeability of ±1% and a nonlinearity of ±0.3%. Figure 4 shows the application circuit of the pressure sensor. The netlabel U4-2 represents the pressure sensor, and the netlabel DRIV4 represents the selected drive of the pressure sensor. The signals are amplified by the amplifier circuit with the AD8617 operational amplifier. There were noises in the output signals, and the capacitance of the bypass capacitors could be adjusted to filter them out. In our design, the capacitance of bypass capacitors is 0.1 μ*F*, which can filter out the noises existing in the output signals. The values of resistor and capacitor are 10${}^{5}$ Ω and 0.01 μ*F* respectively. The cutoff frequency of the low-pass filter is calculated to be 15.9 Hz according to Equation (3).

### 2.3. CO${}_{2}$ Concentration Measurement Module

Given the requirements of CO${}_{2}$ concentration monitoring clinically, a CO${}_{2}$ concentration measurement module has been designed based on the Lambert–Beer law:

(2) $${I=I}_{0}e^{-\alpha CL}$$

where I is the intensity of light striking the detector (I, ${W/\mathrm{cm}}^{2}$), $I_{0}$ is the measured intensity of an empty sample chamber ($I_{0}$, ${W/\mathrm{cm}}^{2}$), *α* is the absorption coefficient (*α*, ${\mathrm{cm}}^{2}/\mathrm{mol}$), *C* is the CO${}_{2}$ concentration (*C*, ${\mathrm{mol}/\mathrm{cm}}^{3}$) and *L* is the absorption path length (*L*, cm) [17,27,28].

**Figure 5:**
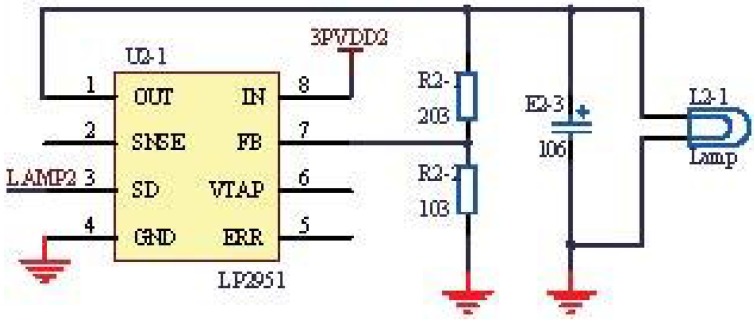
Application circuit of the infrared light source.

As shown in Figure 1, the infrared light-generating source was adopted. Additionally, the infrared light was absorbed by carbon dioxide through the gas chamber. According to the reduction amount, the concentration of carbon dioxide can be obtained. Figure 5 shows the circuit of the infrared light source.

**Figure 6:**
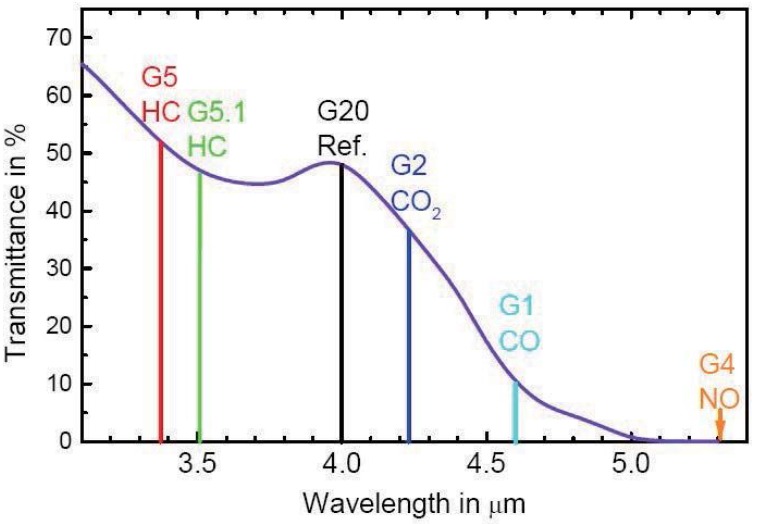
Transmittance of the lamp at the wavelength.

In Figure 5, the netlabel LAMP2 represents the control signal generated by the microprocessor. Additionally, the modulation frequency and depth of the infrared light source can be adjusted by changing the control signal. The lamp can generate a wavelength ranging from 0 to 5.0 μm, according to the datasheet shown in Figure 6, which meets the experiment requirements.

**Figure 7:**
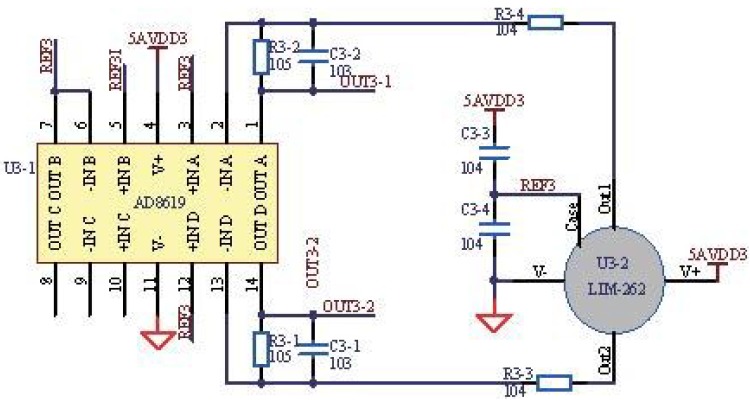
Application circuit of the pyroelectric sensor.

The pyroelectric sensor has a dual channel with a center wavelength and half power bandwidth 4.26 μm/180 nm for the CO${}_{2}$ channel and 3.95 μm/90 nm for the reference channel, respectively. The primary advantage of the pyroelectric sensor is the uniformity of its spectral characteristics [29], and with the advantages of a fast response time, low noise, low input voltage and good stability, it is suitable for continuous and real-time gas detection. In addition, moisture is typically an issue for the pyroelectric sensor, which influences the measurement. In our previous work, we have worked out the moisture issue and adopted the same method [7]. Figure 7 shows the application circuits of the pyroelectric sensor, including the filter circuit and the amplifier circuit with the AD8619 operational amplifier. The benchmark signal is produced by the digital analog converter (DAC) of the microprocessor, as shown in the netlabel REF3, and the output signals of the pyroelectric sensor are amplified based on the benchmark.

## 3. Methods

### 3.1. Power Analysis of the Circuits

Two kinds of sensors are used for the biomedical device, and both need a stable power source. Reliable and continuous power supply for the sensors, microprocessor and signal amplifiers used in a biomedical device are essential for the healthcare of patients [1]. We improved the power circuits and employed some denoising methods.

**Figure 8:**
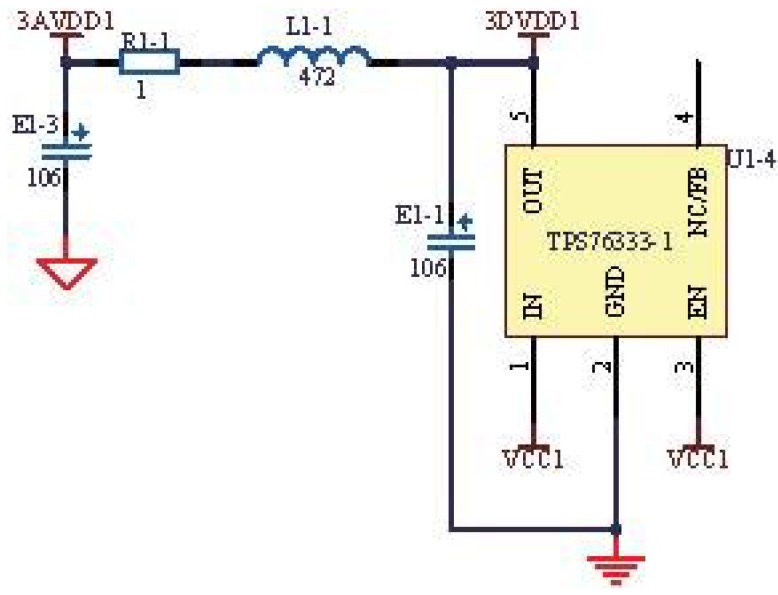
Isolated circuit of power.

All of the circuits include analog power and digital power. To prevent the analog power from being affected by the digital power, we adopted isolated power, as shown in Figure 8.

The circuit in Figure 8 is the typical structure of *π*, which isolates the analog power and digital power. The capacitors E1-1 and E1-3 can filter out the high-frequency signal, and the resistor R1-1 plays the role of over-voltage protection, while the inductance L1-1 can allow the DC (direct current) to pass and hold back the AC (alternate current). Through these settings, the power sources become stable.

Additionally, the AGND (analog ground) and DGND (digital ground) are also isolated by the resistor, rather than the inductance. The characteristic of the inductance is not stable, and the parameters are the least controllable. Therefore, we used the resistor with a resistance of 0, which can ensure the same DC and weaken the noises in some frequencies. The DGND is sensitive to the voltage drop caused by the conductor resistance, and the AGND is not sensitive to the voltage drop; so, the DGND is connected to multiple points to eliminate the voltage drop, and the AGND is connected to a single point.

There exist some noises in the circuit, so the filter circuit is adopted as shown in Figure 7. The cutoff frequency is determined by the resistor and the capacitor. The relationship can be described as follows:

(3) $$f_{c}=\frac{1}{2\pi RC}$$

The capacitor C plays a key role in filtering noise. Given that the frequency of the noise in the environment was at most 50 Hz, we adjusted the resistance of the resistor and the capacitance of the capacitor to filter out the noise. The resistance of R is 10${}^{5}$ Ω, and the capacitance of C is 0.01 μ*F*; then, according to Equation (3), the cutoff frequency $f_{c}$ is calculated to be 15.9 Hz, which fulfills the requirement.

### 3.2. Fluctuation Analysis of Sensors

**Figure 9:**
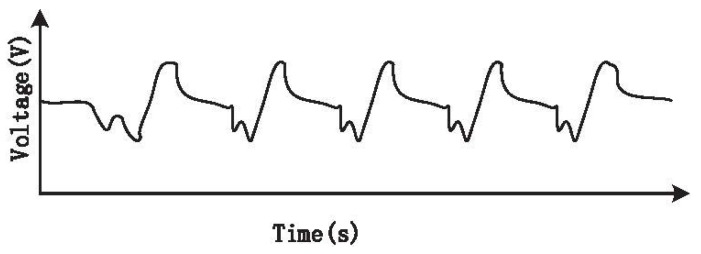
CO${}_{2}$ sensor signal before eliminating fluctuation.

When doing experiments to acquire CO${}_{2}$ concentration with the biomedical device, we found that the signal waveform was not accurate. At the trough of the waveform, there was a small crest periodically (see Figure 9). These small fluctuations will influence the monitoring of CO${}_{2}$ concentration.

The CO${}_{2}$ concentration monitoring module mainly consists of two components, namely a pyroelectric sensor and an infrared light source. First, the infrared light source works by modulation technology, and the power of the drive chip of the infrared light source and that of the pyroelectric sensor are supplied by the same power source. Through experiments, we found that there was a relationship between the fluctuations of the signal and the voltage source, and it was found that the modulation signal changed the voltage source, causing the fluctuations of the output of the pyroelectric sensor. In order to eliminate the fluctuations, the pyroelectric sensor and the infrared light source are powered separately. Additionally, this is also related to the modulation signal. The modulation signal is related to the modulation parameters. We chose the sample frequency *F* and the modulation depth *D* to discuss the relationships. The control variable method is used to discuss the influence brought by the two parameters, respectively. Through several experiments and analysis, we determined the optimal values of the two parameters.

Experiments with no air flowing through the airway tube and a stable experimental environment were conducted. During these experiments, we found that the zero output of the two channels of the pressure transducer had small fluctuations, which were different when different voltages were used to drive the pressure transducer. In response to this, we collected the zero output data of the pressure sensor while using different voltages and analyzed the data with mathematical statistics. The direct drive of the pressure transducer was produced by the DAC of the processor. The output of the DAC has a selectable range: 0∼3.3 V. Based on our results, we will choose a voltage value that ensures a stable output.

In order to quantify the stability of the output, the CV(coefficient of variation) was chosen to assess the output.

(4) $$CV=\frac{\sigma }{M}$$

where *σ* is the standard deviation, *M* is the mean value of the collected data. The smaller the CV is, the more stable the output will be. Based on our results, we will choose the optimal drive.

Moreover, we also took the design of the circuit into consideration, so as to ensure that the power source was stable. The voltage follower was adopted in the circuit, so that the output of the DAC has the characteristic of the voltage source.

## 4. Results and Discussion

### 4.1. Analysis of Stable Power

**Figure 10:**
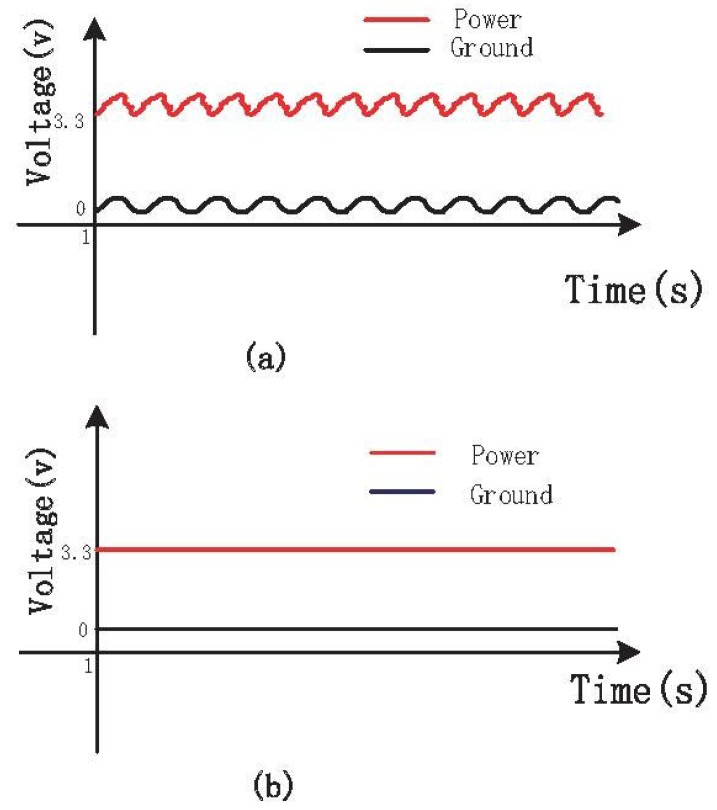
(**a**) Power and ground signals before isolating; (**b**) Power and ground signals after isolating.

Figure 10a shows the measured signals of power and ground containing fluctuations and noises, which influence the respiratory signals. Without the isolated circuits, the power fluctuated up and down about 0.2 V based on 3.3 V, and similarly, the ground fluctuated up and down about 0.2 V based on 0 V. To solve this problem, the isolated circuits were redesigned as shown in Figure 8. After redesigning the circuits, the signals of power and ground are improved greatly, as shown in Figure 10b. Both the power and the ground become smooth and stable, and the fluctuations and noises are eliminated. Additionally, the stable power and ground can help acquire more stable respiratory signals through the following experiments. It can be shown that the isolated circuits will influence the stability of power and ground. In addition, the pyroelectric sensor module, the infrared light module and the pressure sensor module are detachable, which is convenient for replacing the out of order module.

### 4.2. Analysis of the Drive of the Pressure Sensor

The optimal drive was determined with repeated experiments. First, the system was warmed up for 1 min. After warming up, we collected the sensor output at different drives when there was no air flowing. This method was used to collect data for multiple times. Second, we calculated the coefficient of variation of the collected data. Table 1 shows the relationships of the CV values and zero output; CV1 and CV2 represent the zero output of Channel 1 and Channel 2, respectively. By comparison, the values of CV1 and CV2 are the smallest when a drive of 2.5 V is used and no air is flowing, and we can conclude that the best drive is 2.5 V. Using a drive of 2.5 V, the zero output will be more stable and the signals will be more accurate.

**Table 1:** The coefficient of variation at different drives.

Voltage (V)	1 V	1.5 V	2 V	2.5 V	3 V	3.3 V
*CV* 1	0.00091	0.000802	0.000918	0.000637	0.001046	0.000961
*CV* 2	0.001479	0.001086	0.001124	0.000838	0.001206	0.002249

**Figure 11:**
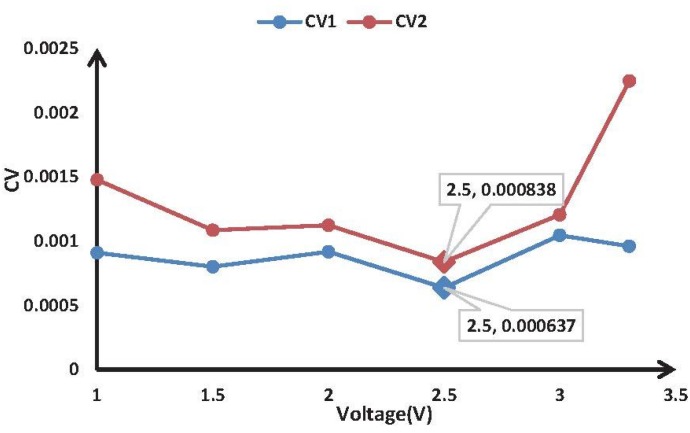
Relationship between the drives and the CV values.

Figure 11 shows the relationship between the drives and the CV values according to Table 1 and displays the result more intuitively for readers.

### 4.3. Analysis of Power Consumption

The power consumption of whole circuits mainly is the power cost of the infrared light source. The infrared light source works at a voltage of 3.3 V and a current of 0.12 A, and so, the total power consumption of the infrared light source is 0.396 W. In order to measure CO${}_{2}$ concentration accurately, the modulation technology was used to modulate the infrared light, and the duty cycle of the modulation technology is 50%. Therefore, the actual power of the infrared light in the device source is 0.198 W. The microprocessor adopted has a power cost of 0.12 W. In addition, the sensors adopted are low-power sensors, and the total power cost of the two sensors is less than 0.01 W. Additionally, through the actual measurement, the power consumption of the device is 0.586 W, which makes sure that the device has the advantage of low power.

### 4.4. Analysis of Stability of Device

**Figure 12:**
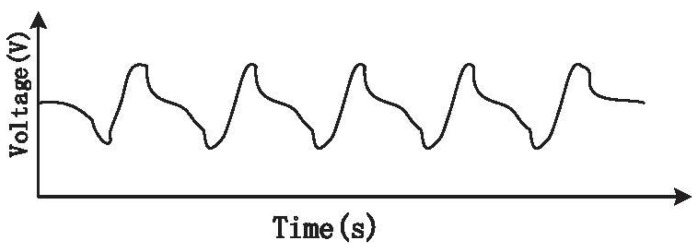
CO${}_{2}$ sensor signal after eliminating fluctuation.

The modulation parameters T=180 ms and D=50% were selected in the system to eliminate the fluctuations at the trough of the waveform, as shown in Figure 12 [7]. Additionally, the drive of the pressure sensor was chosen as 2.5 V according to Section 4.2. After these settings, we chose 20 healthy volunteers as participants for testing. Before testing, they were asked to calm down, but not to consciously control their breathing.

The values of respiratory signals were collected to test the stability of the device. A simple normalization method was adopted. The reference condition was breathing, and the CO${}_{2}$ and flow were normalized by reference sensors. We chose 20 healthy volunteers to do the stability test. The biomedical device was connected to the standard CO${}_{2}$ concentration detector DR95C-CO${}_{2}$-IR and flow detector MF5700. Both of the standard detectors have high accuracy in calibration. We collected the data of the device and the standard detectors, respectively. The data of the standard detectors were chosen as the norm of the normalization. Additionally, then, through the normalized analysis, we got the stability test result. These values were normalized according to equation:

(5) $$N_{i}=\frac{S_{i}}{\frac{1}{n}\sum_{i=1}^{n}S_{i}}$$

**Figure 13:**
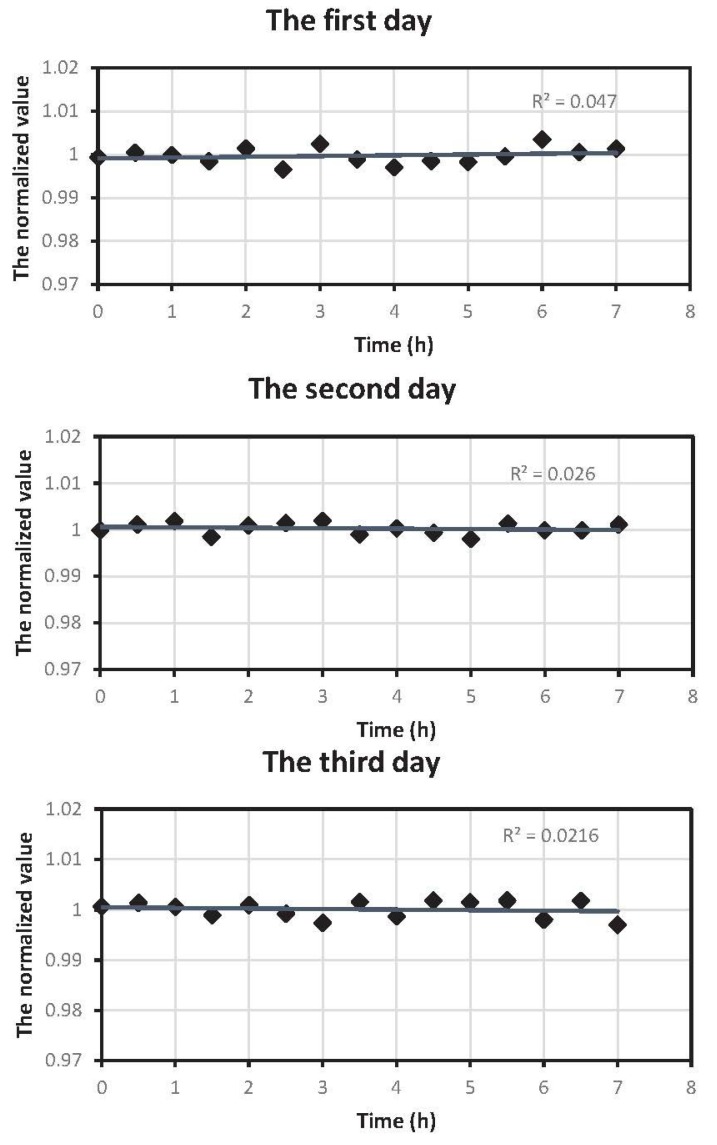
Stability test of the device.

Figure 13 shows the change trend of the normalized values of respiratory signals, including gas flow and CO${}_{2}$ concentration, during three days of continuous testing. The device is being powered by a single power supply at a voltage of 6 V during the experiment, and the power supply is stable and has good performance. Additionally, the 5 V or 3.3 V voltage is acquired by the voltage chip to supply the different circuits inside the device, which makes sure that the power supply is more stable. Additionally, then, we began to do the stability test, and the goal was to demonstrate the reproducibility with a stable power supply. It can be inferred that the normalized values are within 0.99 to 1.01, indicating that the device is stabler, which also satisfies the requirement of continuously working in the long term.

**Figure 14:**
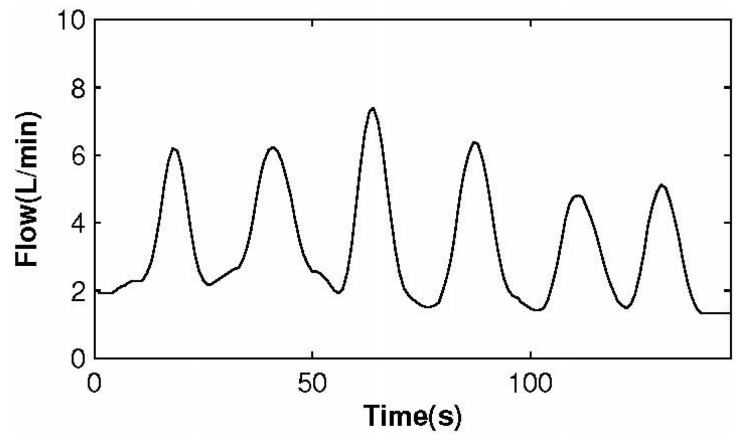
Respiratory flow signal.

**Figure 15:**
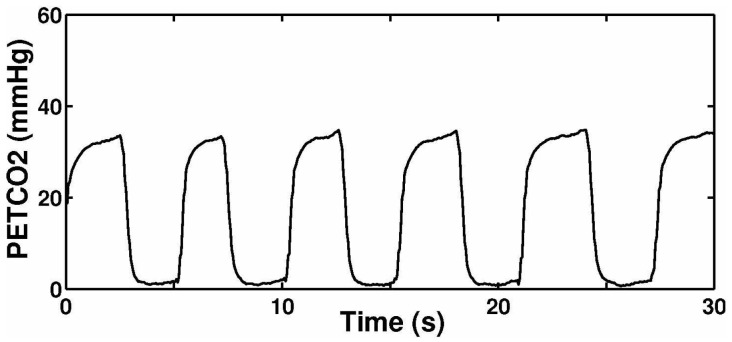
Respiratory CO${}_{2}$ signal.

Figure 14 illustrates the measured gas flow signal sampling from participants with the biomedical device. Figure 15 shows the signal of measured CO${}_{2}$ concentration. The results summarized from Figure 14 and Figure 15 demonstrated that the biomedical device can accurately monitor respiratory gas flow and CO${}_{2}$ concentration simultaneously. Compared with other devices reported in the literature or commercially, such as the products manufactured by PHASEIN Medical Technologies, the biggest advantage is that the device proposed can realize simultaneous the measurement of gas flow and CO${}_{2}$ concentration.

## 5. Conclusions

In recent years, in the wake of the development of biomedicine, biomedical sensor technology and computer science, biomedical devices have been widely used in health monitoring. As a significant tool in clinical monitoring, biomedical devices for respiratory monitoring have been of more concern with respect to researchers and patients.

The current paper presented a detailed introduction of a portable and low-power biomedical device for respiratory monitoring. Respiratory gas flow and CO${}_{2}$ concentration monitoring were considered. Additionally, the biomedical module utilized in the device, the stable power source of the adopted sensors, the design of the circuits and the targeted experiments were summarized. Currently, with the device, we have been able to collect respiratory signals from the participants. Therefore, the methods or techniques adopted in this paper ensure that the device can work continuously for a long time. The proposed biomedical device can be used to accurately monitor the respiratory gases of patients in a clinical environment. On the other hand, we shall further develop the significant application prospects for the respiratory monitoring and the related techniques.

Until now, respiratory monitoring has made great progress and shown good application prospects. Biomedical devices in the future in this field can realize more parameters for measurement based on our device. With progress in developments, such as analytical algorithms, miniature sensing modules and miniature integrated platforms, stable power supplies, and so on, the device for respiratory monitoring will become easy and effective and will be widely used in daily life, as well as various clinical situations.
